# Plant-derived extracellular vesicles as emerging biotherapeutic agents and delivery vehicles for rheumatoid arthritis: evidence from preclinical models

**DOI:** 10.3389/fimmu.2026.1840203

**Published:** 2026-05-08

**Authors:** Yun Liu, Xiaoyu Xu, Na Zou, Qing Chen, Sitong Wu

**Affiliations:** 1 School of Pharmacy, Liaoning Vocational College of Medicine, Shenyang, China; 2 School of Basic Medicine, Shenyang Medical College, Shenyang, China; 3 Shenyang Key Laboratory of Medical Molecular Theranostic Probes in School of Pharmacy, Shenyang Medical College, Shenyang, China

**Keywords:** engineering strategies, nanomedicine, plant-derived extracellular vesicles, therapeutic mechanisms, translational challenge

## Abstract

Plant-derived extracellular vesicles (PDEVs) are emerging as promising natural nanotherapeutics for rheumatoid arthritis (RA). This review summarizes the therapeutic potential of PDEVs, highlighting their unique biological properties, multi-target mechanisms of action, and current application challenges. Accumulating evidence indicates that PDEVs can modulate immune responses, suppress inflammatory pathways, exert antioxidant effects, protect bone and cartilage, and influence the gut-joint axis. Meanwhile, engineering strategies, including drug loading, targeted modification and integration with smart materials, significantly enhance their therapeutic precision and stability. Despite their favorable biocompatibility and cross-barrier delivery potential, challenges such as insufficient standardization of isolation protocols, product heterogeneity, and limited mechanistic insight continue to hinder clinical translation. To date, the majority of studies have been conducted in cell culture or animal models, and clinical data remain unavailable. Future efforts should focus on standardization, in-depth mechanistic studies, and rigorous preclinical validation to accelerate clinical translation for RA and related inflammatory diseases.

## Graphical Abstract

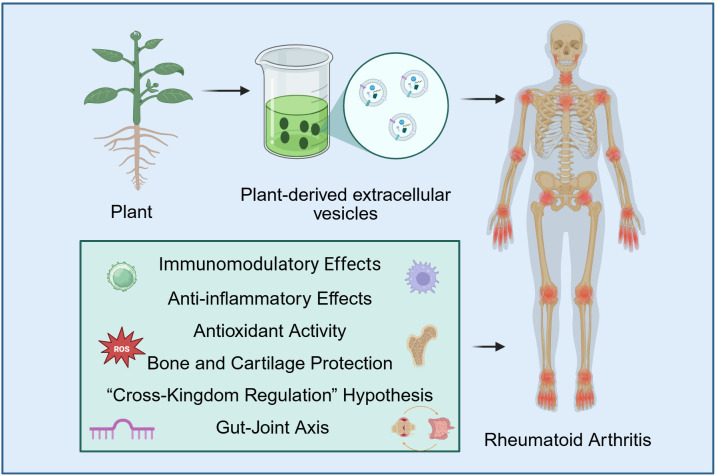

## 1 Introduction

RA is a chronic, systemic autoimmune disease that primarily affects the synovial joints, leading to progressive joint destruction, functional disability, diminished quality of life, and increased mortality (1, 2). As of 2021, RA affects an estimated 17.9 million people globally, representing a significant public health challenge. The disease also demonstrates a clear gender disparity, with a higher prevalence observed among women (3). The clinical burden and epidemiological profile of rheumatoid arthritis are intrinsically linked to its complex pathological mechanisms.

**Figure 1:**
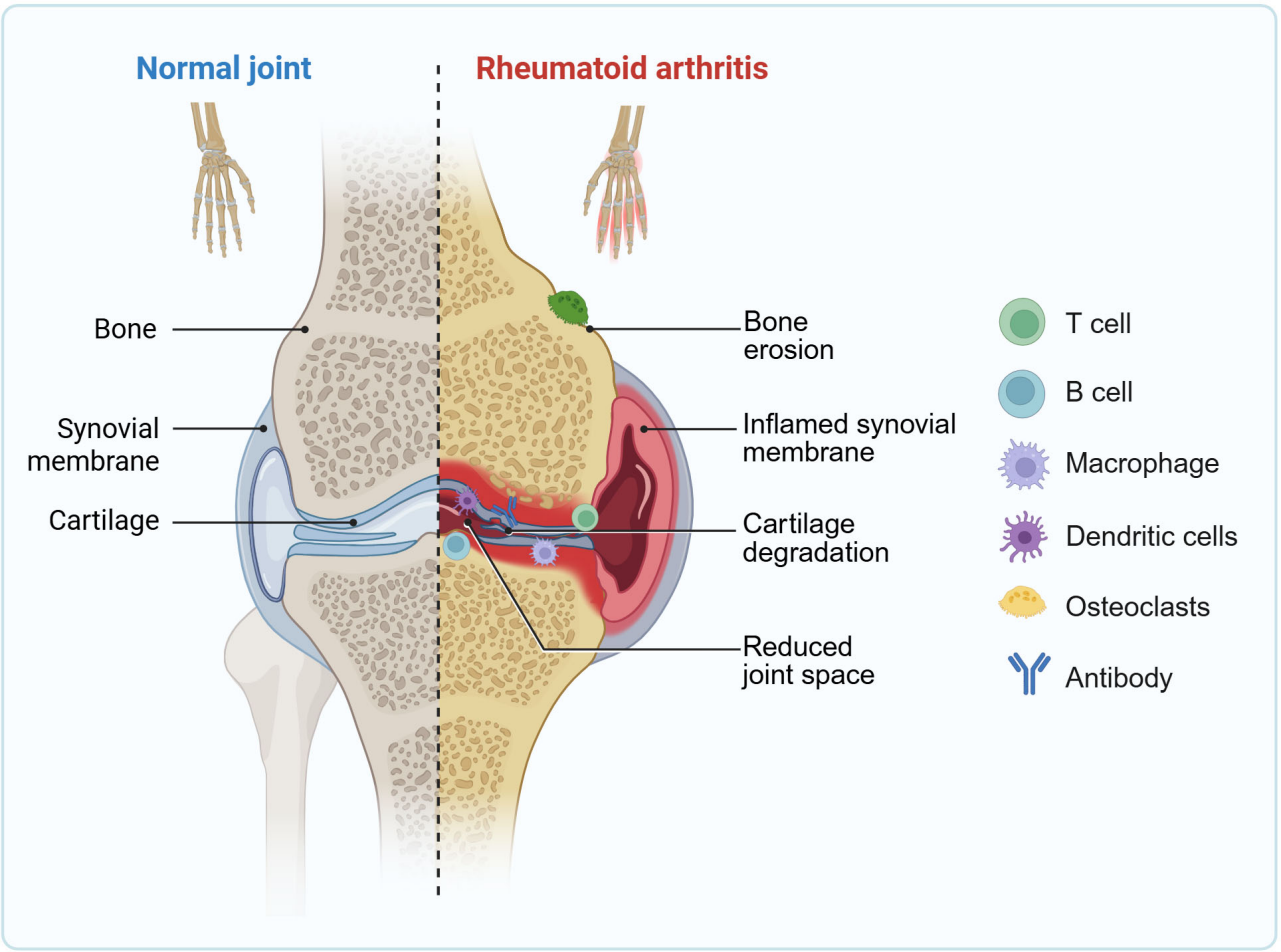
Schematic comparison of joint architecture in health and RA. Normal joint: Displays intact anatomical structures with a thin, non-inflamed synovial membrane. Rheumatoid arthritis joint: Illustrates the characteristic pathological alterations, including marked synovial hyperplasia and inflammation, infiltration of immune cells, the presence of antibodies, and the activation of osteoclasts leading to bone erosion and cartilage destruction. Created with BioRender.com, accessed on January 9, 2026.

Pathologically, RA is characterized by chronic synovitis and progressive joint destruction. The interplay between genetic susceptibility and environmental factors, such as smoking, triggers an autoimmune response, leading to the production of autoantibodies against citrullinated proteins, including anti-citrullinated protein antibodies. These immune complexes activate the complement system and promote the infiltration of innate and adaptive immune cells, such as macrophages, T cells, and B cells, into the synovium (**Figure 1**) (4–6). Immune cell hyperactivation triggers a surge of pro−inflammatory cytokines, such as IL-17, IL-1β, IL-6, and TNF-α, creating a “cytokine storm”. This not only sustains synovial inflammation but also critically activates resident fibroblast-like synoviocytes (FLS) (4, 7). In the RA microenvironment, FLS undergo pathological transformation, acquiring invasive, anti-apoptotic, and pro-inflammatory properties. They proliferate extensively to form pannus and secrete destructive enzymes such as matrix metalloproteinases (MMPs), which directly erode articular cartilage and subchondral bone, ultimately resulting in joint deformity and loss of function (7, 8). Moreover, persistent systemic inflammation increases the risk of multi-organ complications in RA patients, including cardiovascular diseases and interstitial lung disease (9, 10).

Current management of RA is centered on early diagnosis and intensive intervention guided by a “treat-to-target” strategy, which has become the cornerstone for improving long-term outcomes (4). The therapeutic arsenal for RA has undergone a substantial expansion. It has evolved from conventional synthetic Disease-Modifying Anti-Rheumatic Drugs (csDMARDs) to include agents that target specific pathological pathways, such as biologic DMARDs (bDMARDs) against tumor necrosis factor-α or interleukin-6. More recently, the arsenal has been further augmented by oral targeted synthetic DMARDs (tsDMARDs), including Janus kinase (JAK) inhibitors (11, 12). Despite these advances, the clinical management of RA continues to face several significant challenges. First, a substantial proportion of patients exhibit an inadequate response to existing therapies, progressing to “difficult-to-treat RA” (D2T-RA). D2T-RA is defined as the failure to achieve treatment targets despite csDMARD therapy and subsequent treatment with at least two b/tsDMARDs of different mechanisms of action (13). Recent studies indicate that the prevalence of D2T-RA can be as high as 22.3 per 100 among patients exposed to b/tsDMARDs (14). Second, safety concerns associated with advanced therapies remain a significant issue. For example, a large-scale safety trial showed that JAK inhibitors had a higher risk of major adverse cardiovascular events, malignancies, and thromboembolism compared to TNF inhibitors. This finding led to stringent regulatory warnings regarding their use (15). Furthermore, a significant “precision medicine gap” persists in RA care. Currently, there is a lack of clinically validated biomarkers to predict an individual patient’s optimal therapeutic response to a specific targeted agent. This absence means treatment choices often depend more on trial and observation than on a clear understanding of the underlying molecular profile (16, 17). Therefore, there is a clear and urgent need to develop novel therapeutic strategies that are safe, effective, and capable of addressing current therapeutic limitations. Ideally, future approaches would aim to modulate multiple targets, addressing the complex pathological network of RA more comprehensively.

Extracellular vesicles (EVs) represent a novel class of cell-free therapeutic agents, capable of mediating intercellular communication by delivering bioactive cargo such as proteins, lipids, and nucleic acids (18), and they can be derived from both animals and plants. Among them, EVs derived from animal (including human) cells have attracted considerable attention. In particular, mesenchymal stem cell-derived EVs have become a research focus in RA therapy due to their demonstrated immunomodulatory and tissue-reparative properties in preclinical models (19–22). However, the application of EVs faces several inherent challenges. First, high production costs are associated with complex cell culture systems. Second, the lack of standardized isolation protocols leads to heterogeneity and batch-to-batch variability. Third, there are potential immunogenicity and ethical concerns due to their animal or human origin (23–28). Against this background, a subclass of EVs derived from plant sources, known as PDEVs, has emerged as a novel and promising alternative. Belonging to the EV family, PDEVs possess comparable structure and function to mammalian EVs, yet benefit from the inherent advantages of a plant origin: abundant sourcing, low cost, and high biocompatibility (29–33). Their low immunogenicity and remarkable “cross-kingdom communication” capacity enable PDEVs to serve as efficient delivery vehicles, transporting functional small RNAs and proteins into mammalian cells to modulate inflammatory and immune pathways (29–31). Accumulating evidence suggests that PDEVs hold great potential in regulating the immune microenvironment and suppressing inflammation, offering a novel and natural therapeutic strategy for inflammatory diseases such as RA.

Previous reviews on PDEVs have mainly focused on general inflammatory diseases or only briefly mentioned RA (34, 35). In contrast, reviews of EVs in RA have primarily centered on mammalian sources (36, 37). Thus, a systematic synthesis of PDEVs specifically for RA is still lacking. The present review fills this gap by providing an integrated framework that covers the biological properties of PDEVs, their multi-target mechanisms in RA, engineering strategies to enhance their performance, and current translational challenges. Specifically, the review begins by outlining the sources, isolation methods, physicochemical properties, and biocompatibility of PDEVs. It then discusses their therapeutic potential and mechanisms of action, including immunomodulation, anti-inflammation, antioxidant effects, cartilage protection, gut-joint axis modulation, and cross-kingdom regulation. Subsequently, current challenges and engineering strategies are analyzed. Finally, the clinical translation prospects of PDEVs-based therapies for RA are presented.

## 2 Biological properties and preparation of PDEVs

### 2.1 Sources and isolation

PDEVs are a class of bioactive nanoscale vesicles. Their research history can be traced back to 1967, when such vesicular structures were first observed in plant cells (38). In recent years, with the deepening understanding of EVs, PDEVs have garnered increasing attention due to their potential applications in areas such as anti-tumor activity, immunomodulation, and tissue regeneration.

**Figure 2:**
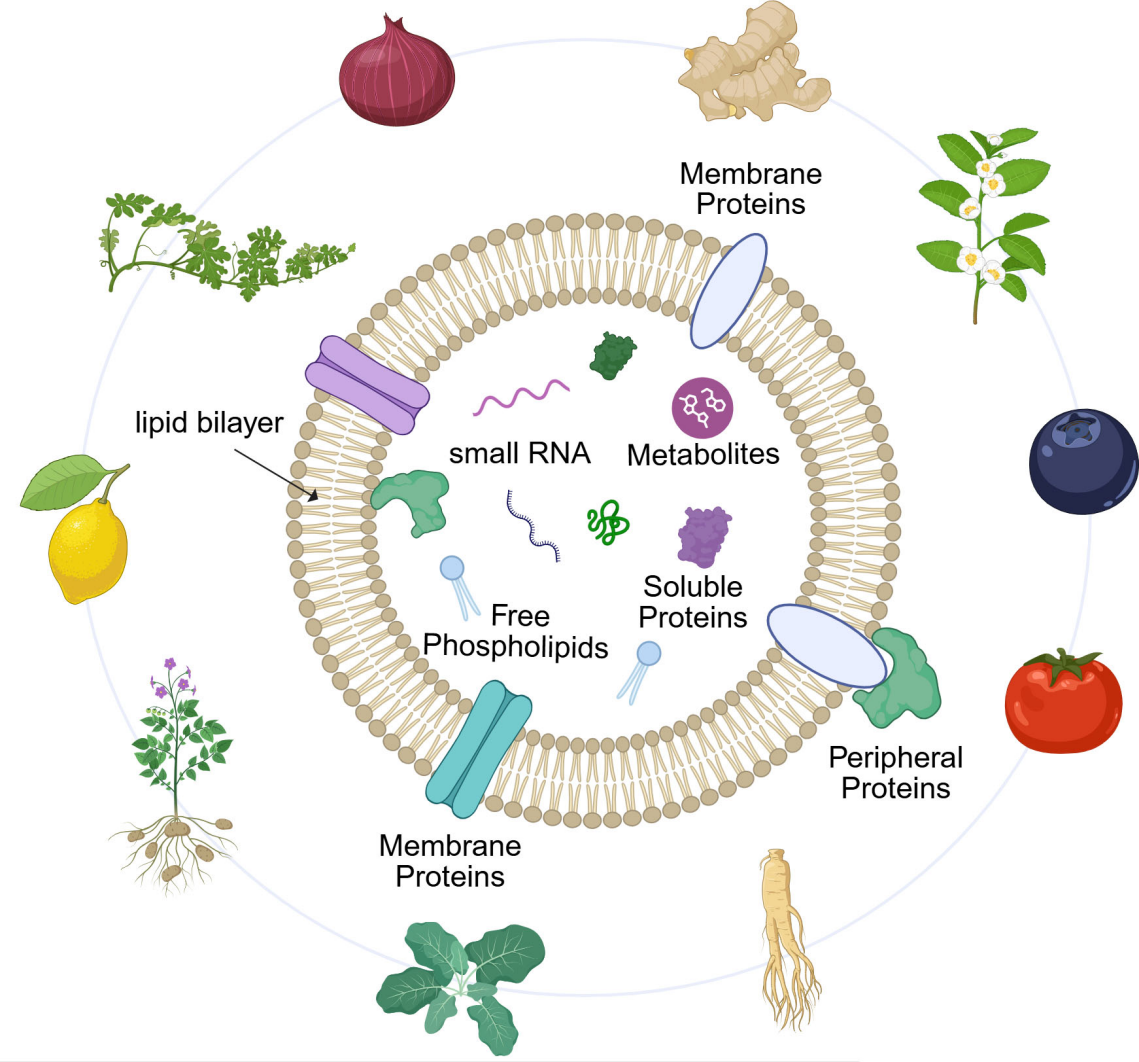
Schematic diagram of the basic structure and main components of PDEVs. The illustration depicts a spherical PDEV bounded by a lipid bilayer in which membrane proteins are embedded. The vesicle lumen contains representative cargo molecules, including small RNAs and metabolites. Surrounding the vesicle are commonly co-isolated non-vesicular components: free phospholipids, soluble proteins, and peripheral proteins. Created with BioRender.com, accessed on January 9, 2026.

PDEVs are sourced from diverse everyday fruits, vegetables, and medicinal herbs (**Figure 2**) (39). This characteristic confers a significant advantage over animal-derived EVs: their production does not rely on complex cell culture systems but rather on direct extraction from abundant, renewable, and low-cost plant biomass, endowing them with great potential for scalable manufacturing (40). To date, studies have confirmed that numerous plants are excellent sources of PDEVs, such as ginger, grapefruit, broccoli, blueberries, tomatoes, shiitake mushrooms, dandelion, tea, and honey (41). Importantly, the therapeutic potential of PDEVs is intrinsically linked to their plant origin, which dictates their unique bioactive cargo and, consequently, their functional properties. For instance, PDEVs from different plants exhibit distinct and specialized functions, such as anti-tumor or anti-inflammatory activities, a diversity rooted in their source-specific molecular compositions (42–45). This inherent “functional diversity” positions PDEVs as a versatile platform for developing multi-target therapies against complex diseases like RA.

The isolation of PDEVs from complex plant materials, while preserving their structure and bioactivity, is a critical step for research and application. However, due to their natural differences in size, composition, and function, a standard isolation method is still lacking (34). As summarized in **Table 1**, a variety of isolation methods used for different plant sources result in a broad spectrum of particle size distributions for the extracted vesicles.

**Table 1:** Common isolation methods and size characteristics of PDEVs.

Plant source	Size range (nm)	Isolation method key steps	Ref
Tea	~131 ~140	Differential centrifugation; Sucrose gradient ultracentrifugation.	(42) (43)
Ginger	~70	Differential centrifugation; Ultracentrifugation	(44)
	156 ± 36	Differential centrifugation; Commercial exosome isolation kit	(45)
	~100	Differential centrifugation; PEG 6000 precipitation.	(46)
Grapefruit	120 – 170	Differential centrifugation; Size Exclusion Chromatography	(47)
	~241	Differential centrifugation; Sucrose gradient ultracentrifugation.	(48)
Ginseng	92.0 ± 4.8	Differential centrifugation; Sucrose gradient ultracentrifugation.	(49)
	144.1 – 340.1		(50)
	176.2 ± 4.6		(51)
*Perilla frutescens*	98.4 ± 1.3	Differential centrifugation; Sucrose gradient ultracentrifugation.	(52)
	130 – 200		(53)

Differential Ultracentrifugation is the most classical method, separating particles based on differences in their size and density within a gradient (41). This method is straightforward and does not require the introduction of exogenous chemical reagents. However, its limitations are notable. The process is time-consuming and demands high-end equipment. Under extreme centrifugal forces, it can easily induce irreversible vesicle aggregation and membrane damage. It also causes co-precipitation with non-vesicular protein contaminants. These issues lead to compromised functional activity and reduced recovery rates (47, 54, 55). It is noteworthy that the optimization of centrifugation parameters is crucial. For *tomato-derived PDEVs*, 100,000 × *g* effectively enriched vesicles rich in proteins and miRNAs, whereas higher forces (120,000 × *g* or 140,000 × *g*) yielded smaller, membrane-deficient particles with reduced bioactivity (41). To address purity issues, density gradient ultracentrifugation, typically using sucrose, is employed as a subsequent refinement step. In this method, the pre-enriched sample is subjected to ultracentrifugation over a pre-formed sucrose gradient. Vesicles migrate to and band at their specific buoyant density, effectively separating intact PDEVs with preserved lipid bilayers from contaminants of differing densities, such as protein aggregates. This yields high-purity vesicles suitable for precise functional and omics analyzes (43, 56). Consequently, a sequential combination of “Differential Centrifugation” followed by “Density Gradient Ultracentrifugation” has been established as a standard strategy in the field, where the former enables rapid initial enrichment and the latter achieves high-purity isolation (42, 57).

Polymer-Based Precipitation is widely adopted due to its low cost, simplicity, and capacity to process large sample volumes (58). However, this method suffers from insufficient specificity, resulting in limited product purity and often necessitating additional steps to remove co-precipitated impurities. Size-Exclusion Chromatography offers a gentler physical separation route. It relies on gravitational flow to separate particles based on their hydrodynamic volume, which better preserves vesicle structural integrity and offers high experimental reproducibility. Its main drawbacks include limited throughput and being time-consuming (59). High-Selectivity Separation Techniques, such as Immunoaffinity Capture, achieve high-purity isolation by utilizing specific antibodies to recognize marker proteins on the vesicle surface. For instance, antibodies can be designed to target specific plant exosome surface antigens. However, its application faces three major challenges: insufficient research on plant PDEV surface markers, the high cost of ligands such as antibodies, and complex operational procedures (60, 61). Aqueous Two-Phase Systems, such as PEG/dextran systems, represent an emerging method that enables rapid and cost-effective separation by modulating the partition coefficient of vesicles between the two phases. This eliminates the need for ultracentrifugation steps and thus shows promising application prospects. However, potential interference from residual polymers in subsequent analyzes and applications must be considered (62). Furthermore, Microfluidic Technology, leveraging its advantages of speed, high integration, and low sample consumption, demonstrates potential in the separation and detection of PDEVs. Currently, this technology is more suitable for analytical purposes and small-scale preparation, while still facing significant challenges in large-scale, standardized production applications (60).

In summary, the selection and optimization of isolation techniques directly determine the characteristics of the obtained PDEV preparations, thereby influencing subsequent research on biological functions and therapeutic outcomes. Presently, no single method achieves optimal performance simultaneously in yield, purity, vesicle integrity, and active component retention, representing the foremost technical bottleneck for both fundamental research and translational applications of PDEVs (34, 63). Future breakthroughs may come from smarter combinations of existing methods, or from new strategies that separate vesicles based on biological activity to enrich RA-relevant components beyond physical uniformity.

### 2.2 Biological composition of PDEVs

PDEVs exhibit a typical vesicular structure enveloped by a lipid bilayer, predominantly appearing spherical or cup-shaped under transmission electron microscopy (**Figure 2**) (64). Their particle size distribution is broad, typically ranging from 30 to 500 nanometers, although the specific distribution is influenced by plant species and isolation protocols (65). This inherent nanoscale dimension and lipid membrane architecture constitute the physical foundation enabling PDEVs to effectively encapsulate and protect their complex bioactive cargo, facilitating intercellular and even cross-kingdom delivery (66). PDEVs possess a complex biochemical profile that includes lipids, proteins, nucleic acids, and plant-specific metabolites, collectively forming the material basis for their diverse biological functions, as shown in **Table 2**.

**Table 2:** Molecular cargos and demonstrated biological functions of different PDEVs.

Plant source	Key molecular cargos	Demonstrated biological functions	Ref
Tomato	1. Proteins: HSP70, HSP80, Tetraspanin-8, Actin, GAPDH; 2. miRNAs: sly-miR159, sly-miR162, sly-miR1919a.	Modulates intestinal cell (Caco-2) viability and proliferation in a subpopulation-dependent manner.	(41)
Tea	1. Lipids: Phosphatidylcholine, Phosphatidylethanolamine 2. Polyphenols: Epigallocatechin gallate, Epicatechin gallate, Epicatechin 3. Proteins: 745 kinds identified	1. Induces dose-dependent cytotoxicity and apoptosis in breast cancer cells; 2. Inhibits cell migration, invasion, and metastasis; 3. Triggers ROS generation and mitochondrial damage; 4. Suppresses primary tumor and lung metastasis *in vivo*; 5. Modulates gut microbiota diversity.	(42)
	1. Lipids: Phosphatidic acid, Phosphatidylglycerol, etc. 2. Polyphenols/Flavones: Gallic acid, Caffeine, etc. 3. Proteins: Various proteins	1. Macrophage-specific uptake via galactose-mediated endocytosis; 2. Antioxidant: Reduces ROS and upregulates HO-1; 3. Anti-inflammatory: Modulates cytokines (TNF-α, IL-6, IL-12); 4. Protects gut barrier: Preserves ZO-1 and MUC2; 5. Prevents/alleviates colitis and colitis-associated cancer.	(43)
Ginger	1. Bioactive Metabolites: 6-Gingerol, 8-Gingerol, 10-Gingerol	1. Efficiently absorbed in the rat small intestine; 2. Serves as a natural nanocarrier with high loading capacity for lipophilic compounds.	(44)
	1. miRNAs: bdi-miR5179, csi-miR396e-5p	1. Anti-inflammatory: Inhibits LPS-induced expression of inflammatory mediators in Caco-2 cells; 2. Internalized via caveolin-mediated endocytosis and micropinocytosis; 3. Cross-kingdom function: RNA-cargo dependent.	(45)
	1. Bioactive Metabolites: 6-Gingerol, 6-Shogaol; 2. Plant miRNAs (hsa-miR-22-3p, miR-143-3p).	1. Target M1 macrophages, promote M2 polarization via PI3K/AKT pathway; 2. Anti-inflammatory: Reduce TNF-α, IL-6, IL-1β; 3. Alleviate synovitis and bone erosion in CIA mice.	(48)
	1. Proteins: ITGB2, HSP70; 2. miRNAs: miR-7263-3p.	1. Targeted immunomodulation; 2. M2 macrophage polarization; Synergistic anti-inflammation; 3. Joint protection in CIA rats.	(46)
Grapefruit	1. Proteins; 2. Nucleic Acids; 3. Other potential antioxidant components.	1. Cartilage Protection and Regeneration: Downregulates catabolic enzyme ADAMTS-5 and hypertrophic marker COL10; upregulates anabolic markers ACAN, COL2, SOX9; 2. Downregulates inflammatory markers COX-2 and PTGS2; 3. Antioxidant: Upregulates antioxidant genes SOD2 and GPX; 4. Promotes Repair: Enhances chondrocyte migration.	(47)
*Arabidopsis thaliana*	1. Diverse proteins 2. Known plant EV markers (PEN1, PATL1, et al.)	1. Efficient uptake by human ovarian cancer cells (OVCAR5); 2. Serves as a foundational method for potential therapeutic delivery.	(55)
Gouqi *(Lycium barbarum)*	1. Lipids; 2. Bioactive Metabolites, such as Terpenoids, Alkaloids, Polyphenols.	1. Promotes osteoblast proliferation and differentiation (MC3T3-E1); 2. Enhances fracture healing in mice (BMD, BV/TV, Tb. Th, OPN, BGP); 3. Activates PI3K/Akt/mTOR/p70S6K/4EBP1 signaling pathway.	(57)
*Morinda officinalis*	Bioactive Metabolites: Anti-inflammatory compounds.	1. Immunomodulation; 2. Bone and Cartilage Protection; 3. Engineering Advantages.	(59)
Ginseng	Lipids: diacylglycerols, phosphatidylcholine, phosphatidylethanolamine, lysophosphatidylcholine, sphingomyelin.	1. Anti-senescence: Reduces SA β-Gal activity in human dermal fibroblasts; 2. Anti-pigmentation: Reduces melanin in UVB-induced senescent melanocytes; 3. Cell viability >80% at 10 µg/mL, safer than crude root extracts.	(49)
	miRNAs: mtr-miR159a, har-miR403a, cca-miR396a-3p.	1. Promotes neural differentiation of BMSCs; 2. Activates PI3K signaling pathway in BMSCs;	(50)
Ginseng	miRNAs: pgi-miR6135j.	1. Oral GDNPs target joints and alleviate arthritis in mice; 2. Inhibit synovial macrophage inflammation (reduce IL-6, TNF-α); 3. Suppress phosphorylation of MAPK pathway (JNK, ERK, p38); 4. Down-regulating KRAS-MAPK signaling.	(67)
	1. Lipids: Ceramides; 2. Other: Ginsenoside Re.	1. Polarizes M2 to M1 via TLR4/MyD88; 2. Recruits CD8^+^ T cells via CCL5/CXCL9 promotes Th1 differentiation.	(68)
Mulberry Bark	Heat shock protein family A (Hsp70) member 8 (HSPA8).	1. Prevents colitis via AhR/COPS8 pathway; 2. Induces antimicrobial peptides & enhances gut barrier.	(69)
*Dioscorea japonica*	Proteins, Small RNAs, Lipids.	1. Stimulates osteoblast differentiation & mineralization *in vitro*; 2. Increases bone density & volume in osteoporotic mice;	(70)
Lemon	Vitamin C, Citrate, Short RNAs.	Antioxidant protection; Osteogenic differentiation promotion; Enhanced collagen synthesis.	(71)
	Galacturonic acid-enriched pectin-type polysaccharide.	Enhances bile resistance of Lactobacilli and downregulates bacterial Msps.	(72)
*Pueraria lobata*	gma-miR4412; Phosphatidic acid.	1. Targets gut–joint axis modulation; 2. Inhibits PEA production and alleviates arthritis in CIA mice via PEA–BTK–NETs axis.	(73)
*Perilla* *frutescens*	Bioactive compounds (such as Rosmarinic acid).	1. Permeation Enhancer: Promotes skin absorption via IL-17-mediated downregulation of tight junction proteins; 2. Anti-inflammatory: Reduces joint swelling and serum IL-6/IL-1α/TNF-α in rat model.	(52)
	1. Nucleic Acids: Small RNAs, specifically pab-miR396a-5p; 2. Metabolites: Enriched in flavonoids and secondary metabolites.	1. Anti-inflammatory and Antioxidant; 2. Immunomodulation: Increases Treg (Foxp3+) cell frequency and suppresses IL-17 signaling; 3. Keratinocyte Modulation: Promote apoptosis; 4. Alleviate psoriasis symptoms in IMQ-induced mouse models.	(53)
Broccoli	1. miRNAs: Endogenous ath-miR159a, ath-miR166b-3p, ath-miR319a, ath-miR403–3p, et al; 2. Protein: Vesicle-associated proteins.	1. Protects encapsulated miRNAs from enzymatic degradation; 2. Delivers functional miRNAs to human intestinal Caco-2 cells; 3. Reduces viability of Caco-2 cells upon delivery of exogenous miRNAs	(74)

Lipids in PDEVs perform both structural and bioactive functions. In addition to forming the vesicle membrane, plant-specific lipids such as phospholipids, sphingolipids, and sterols carry intrinsic bioactivity. For example, ginger-derived PDEVs are rich in lipophilic compounds like gingerols, which exhibit well-defined anti-inflammatory properties (44, 45, 49).

Proteomic studies reveal that PDEVs carry a rich array of proteins. This includes a suite of evolutionarily conserved “housekeeping” proteins involved in fundamental processes such as vesicle biogenesis and trafficking, such as heat shock proteins (HSP70/HSP90), 14-3–3 proteins, and glyceraldehyde-3-phosphate dehydrogenase (GAPDH) (69, 75). They also contain specific proteins reflecting their plant origin, such as the Patellin-3-like protein and clathrin heavy chain identified in citrus PDEVs (76). PDEVs also contain a variety of active enzymes, especially the antioxidant enzyme system, including superoxide dismutase (SOD), catalase, and peroxidase (77). Protected by the lipid bilayer, these enzymes may retain catalytic activity after delivery to mammalian cells, providing a molecular mechanism for directly scavenging excess reactive oxygen species (ROS) in the host.

Nucleic acids are key components mediating the cross-kingdom regulatory effects of PDEVs, with miRNAs being of particular interest. Plant miRNAs exhibit a degree of sequence conservation across species. This enables them to modulate the expression of key genes in pathways related to inflammation, immunity, and apoptosis within mammalian cells through mechanisms such as mimicry or competitive inhibition (78). The lipid membrane of PDEVs effectively protects these miRNAs from degradation, a prerequisite for their oral bioavailability and long-range regulatory effects (79).

Furthermore, PDEVs encapsulate a variety of bioactive secondary metabolites synthesized by the source plants, such as polyphenols, flavonoids, and saponins (80). For example, tea-derived PDEVs are rich in polyphenolic compounds like gallic acid and epigallocatechin gallate, conferring additional antioxidant and anti-inflammatory capacities (43). These metabolites, together with the proteins, RNAs, and lipids within the vesicles, constitute a synergistic library of active molecules.

In summary, the physical and biochemical complexity of PDEVs defines their unique nature. This diversity supports their functional potential, but also complicates standardized research and clinical use. Therefore, comprehensively characterizing PDEVs’ properties is essential to understand their function, assess their therapeutic value, and enable clinical translation.

### 2.3 Biological functions of PDEVs

PDEVs demonstrate broad prospects in the field of drug delivery due to their multiple advantageous biological properties, rendering them particularly suitable for the targeted treatment of chronic diseases such as RA.

Available evidence suggests that some PDEVs possess sufficient stability in the gastrointestinal environment and during long-term storage. For example, PDEVs derived from tea and citrus sources maintain their particle size, surface charge, and structural integrity across a range of pH values (from acidic to alkaline) and in simulated gastric and intestinal fluids (43, 81). Man et al. (44) have further confirmed that ginger-derived PDEVs can be effectively absorbed across various segments of the small intestine (duodenum > jejunum > ileum), indicating their ability to retain functional activity and achieve delivery within the digestive system. Regarding storage stability, research indicates that yam-derived PDEVs can maintain their bioactivity even after one year of storage at -80 °C (70). Furthermore, by employing lyophilization techniques with cryoprotectants such as trehalose, grapefruit-derived PDEVs can preserve their structural integrity and drug encapsulation capability over extended periods at room temperature (47), offering potential for scalable production and clinical application.

PDEVs have been shown in preclinical studies to exhibit favorable biocompatibility and low toxicity, which are advantageous for their potential use as therapeutic carriers, especially for chronic diseases like RA. Their safety stems from the significant evolutionary distance between PDEVs and mammalian systems, as well as their unique lipid and protein composition (such as the complete absence of cholesterol). This greatly minimizes the risk of being recognized as “foreign” by the host immune system and triggering a strong response. Studies confirm that the immunogenicity of PDEVs is typically only 20-40% that of mammalian exosomes (82), establishing a critical safety foundation for their long-term or repeated administration, particularly via the convenient oral route. This characteristic has been systematically validated in numerous studies from *in vitro* to *in vivo* settings. At the cellular level, for instance, turmeric-derived PDEVs show no significant cytotoxicity toward human colorectal adenocarcinoma cells (Caco-2) or mouse macrophages (RAW 264.7) (83). Similarly, ginseng-derived PDEVs (GDNPs) are safe and non-toxic to bone marrow mesenchymal stem cells at concentrations of 0-0.5 µg/mL (50). At the organismal level, studies show that mice receiving continuous oral administration of GDNPs maintain normal body weight and key organ indices. They also maintain crucial serum markers of liver and kidney function (such as ALT, AST, BUN, and creatinine) within physiological ranges, with no signs of toxicity (25). This finding is further supported by research on yam-derived PDEVs, which revealed no histopathological damage in vital organs such as the heart, liver, spleen, lungs, kidneys, and brain in mice following continuous oral administration at therapeutic doses (70). These results collectively demonstrate that oral PDEVs are non-toxic to the body’s primary metabolic and excretory organs. Even in disease treatment contexts, PDEVs exhibit good tolerability. For example, no significant local or systemic adverse reactions were observed following administration of lemon-derived PDEVs in a chronic myeloid leukemia model (71) or tea-derived PDEVs in an inflammatory bowel disease model (43).

PDEVs possess specific targeting capabilities. Liu et al. (55) found that *Arabidopsis thaliana* PDEVs were efficiently taken up by human ovarian cancer cells (approximately 99.5%), indicating cell selectivity. Furthermore, certain PDEVs carry natural surface ligands that facilitate targeted delivery; for instance, galactosyl groups on their surface can bind to galectin receptors on macrophages to promote receptor−mediated uptake (84). This provides a novel strategy for targeting macrophages in RA.

PDEVs have been shown to protect encapsulated drugs from degradation and prolong their circulation time. For instance, PDEVs loaded with small-molecule drugs or nucleic acids exhibit longer half-lives and higher tissue accumulation rates in the circulatory system. Research by Zhang et al. (85) demonstrated that doxorubicin-loaded ginger PDEVs could stably encapsulate the drug via electrostatic interactions and achieve pH-responsive release in the tumor microenvironment, significantly prolonging drug retention at the lesion site. Moreover, surface modifications of PDEVs, such as PEGylation or ligand conjugation, can further delay clearance by the immune system, enabling long-lasting circulation (86, 87).

PDEVs possess the potential to cross biological barriers, particularly the blood-brain barrier, while being unable to traverse the placental barrier. This characteristic enables their potential application in treating central nervous system diseases while ensuring safety during pregnancy. Studies indicate that certain PDEVs can cross the blood-brain barrier via endocytosis, facilitating drug delivery to the brain (88, 89). The same research also noted that these vesicles do not accumulate in placental tissue, indicating favorable pregnancy safety (90). This property suggests that PDEVs may offer advantages for treating central nervous system complications associated with RA, potentially with reduced fetal exposure risks.

Overall, PDEVs exhibit a range of biological functions that support their potential as both intrinsic therapeutic agents and delivery platforms. However, these functions have been demonstrated mainly in a limited number of plant sources and are often based on *in vitro* or non-RA animal models. Moreover, the stability and tissue specificity of PDEVs depend on factors such as plant source, isolation method, and storage conditions. Therefore, the generalizability of these findings to diverse PDEV types and their relevance to the inflamed joint microenvironment in RA patients remain to be systematically evaluated. Future studies should include comparative assessments across multiple plant sources and validation in RA-specific models.

### 2.4 PDEVs for RA treatment

**Figure 3:**
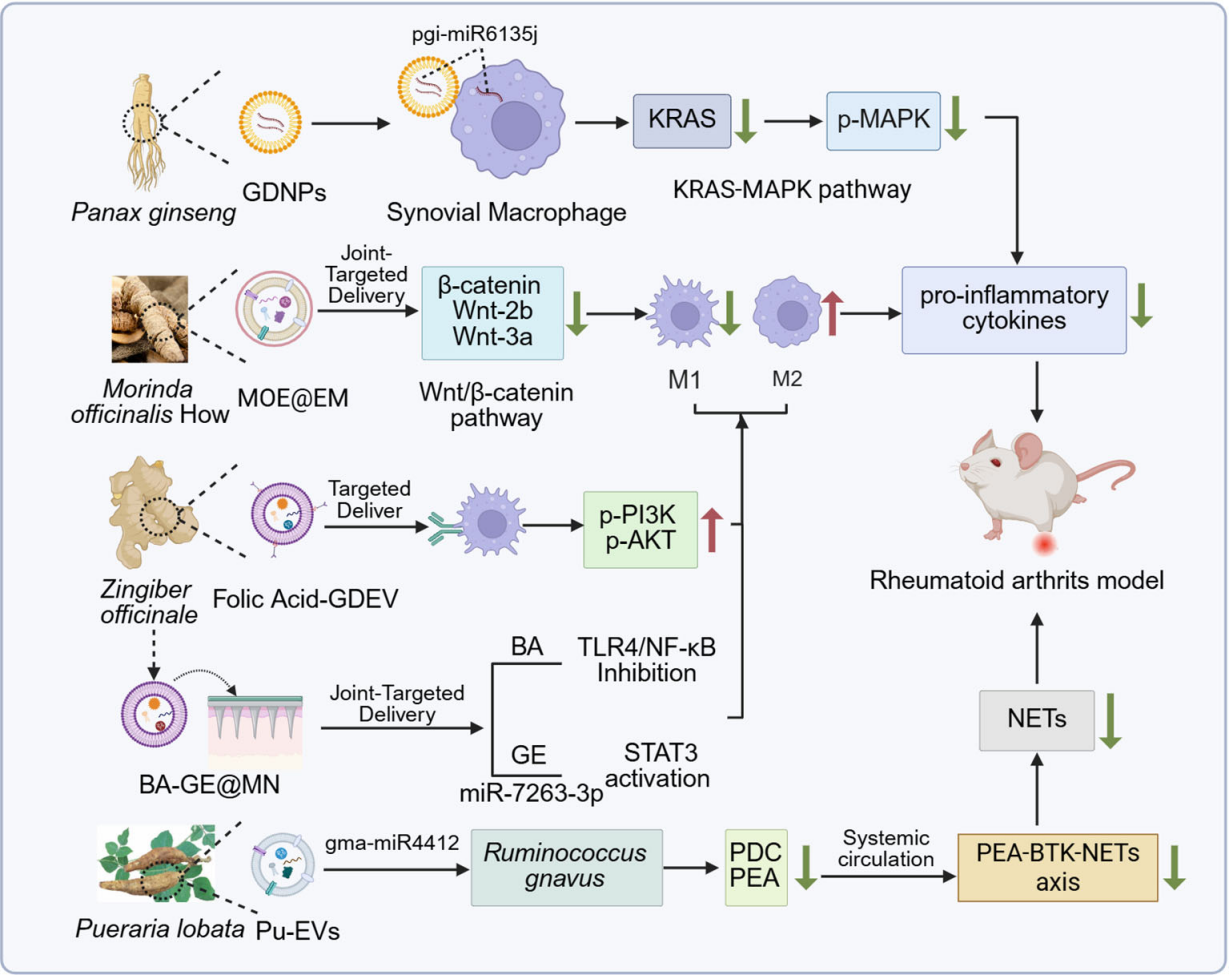
Therapeutic mechanisms of PDEVs from different botanical sources in rheumatoid arthritis models. Created with BioRender.com, accessed on January 9, 2026.

Research on the application of PDEVs in RA therapy has progressed to mechanistic exploration and efficacy validation. Multiple studies utilizing PDEVs from various plant sources have revealed their therapeutic potential and mechanisms of action in arthritis models (**Figure 3**).

Wang et al. (67) confirmed that orally delivered GDNPs selectively accumulate in arthritic joints, markedly reducing joint swelling and mitigating bone erosion in a collagen−induced arthritis murine model. Mechanistic investigation revealed that these PDEVs are internalized by synovial macrophages. They carry the plant miRNA pgi-miR6135j, which directly targets and suppresses the expression of host cell KRAS mRNA. This leads to the downregulation of phosphorylation levels in the JNK, ERK, and p38 components of the MAPK signaling pathway, ultimately reducing the production of pro-inflammatory cytokines such as IL-6 and TNF-α.

Zhang et al. (59) isolated PDEVs from *Morinda officinalis* (MOE) and employed erythrocyte membrane camouflage to construct an MOE@EM delivery system, enhancing joint accumulation via the EPR effect. MOE drives macrophage repolarization from M1 to M2 phenotype by inhibiting the Wnt/β-catenin pathway, significantly reducing paw swelling, synovial hyperplasia, and bone erosion in AIA mice. The study also introduced an ultraviolet irradiation pretreatment strategy, which increased MOE yield by 2–3 fold while enhancing its bioactivity.

Ginger-derived PDEVs (GDEVs) have been engineered for active targeting. A recent study constructed FA-GDEVs by conjugating folic acid to GDEVs, enabling specific uptake by M1 macrophages in inflamed joints via folate receptors (48). FA-GDEVs drive M2 polarization through PI3K-AKT activation and show superior efficacy in alleviating arthritis compared to unmodified GDEVs and methotrexate, highlighting the synergistic advantage of PDEVs as multicomponent nanocarriers. The therapeutic potential of GDEVs can be further enhanced through innovative delivery systems. Han et al. (46) further developed a transdermal “exosome-microneedle” platform by co-loading ginger EVs (GE) and Bullatine-A into dissolvable microneedles (BA-GE@MN). In a rat CIA model, BA-GE@MN delivery reduced paw swelling and serum pro-inflammatory cytokines through synergistic mechanisms: GE promotes M2 polarization via miR-7263-3p, while BA inhibits the TLR4/NF-κB pathway, suggesting a potentially promising local treatment strategy for RA that needs further investigation.

Han et al. (73) further revealed a mechanism by which PDEVs systemically regulate RA progression through modulation of the “gut–joint axis”. Their study demonstrated that *Pueraria lobata*-derived PDEVs (Pu-EVs) can alleviate rheumatoid arthritis by targeting gut microbiota metabolism. Clinical cohort analysis combined with animal experiments showed that *Ruminococcus gnavus* is enriched in RA gut microbiota and promotes neutrophil extracellular trap formation via phenylethylamine secretion, exacerbating arthritis. Mechanistically, Pu-EVs are selectively taken up by *R. gnavus* and deliver the plant miRNA gma-miR4412. This miRNA suppresses bacterial phenylalanine decarboxylase expression, reduces PEA production, and thereby inhibits the PEA-BTK-NETs axis, alleviating joint inflammation. The study also identified phosphatidic acid enrichment as a key lipid feature enabling gut retention and microbiota targeting of Pu-EVs, in contrast to phosphatidylcholine-rich EVs, which exhibit broader systemic distribution.

Studies of their therapeutic mechanisms in related inflammatory models also provide insights translatable to RA. For example, in an acute gouty arthritis model, perilla leaf-derived PDEVs were shown to enhance local drug delivery by modulating the IL-17 pathway and downregulating keratinocyte tight junction proteins (52). Given the established role of IL-17 in driving RA synovitis and bone destruction, these findings imply that PDEVs could not only facilitate drug penetration into inflamed joints but also directly intervene in key pathogenic signaling within the RA microenvironment. In LPS-induced RAW264.7 model, GDNPs dose-dependently inhibited the secretion of key pro-inflammatory cytokines (TNF-α, IL-1β, IL-6). They also significantly downregulated the expression of inflammation-associated enzymes (COX-2 and iNOS) and the production of nitric oxide (51). These molecular-level anti-inflammatory effects precisely correspond to the core pathological processes of RA, providing underlying mechanistic support for the overall therapeutic efficacy observed in the aforementioned animal models.

Collectively, these preclinical studies demonstrate the therapeutic potential of PDEVs in arthritis models. However, the evidence is derived predominantly from small-animal studies with acute or induced arthritis, and direct translation to human RA remains to be established. The reported mechanisms, particularly miRNA-mediated effects, require validation in more physiologically relevant models.

## 3 Potential mechanisms of PDEVs in RA treatment

Current research has preliminarily revealed multiple potential mechanisms by which PDEVs may contribute to RA treatment, primarily encompassing immunomodulation, anti-inflammation, antioxidant effects, and bone/cartilage protection. These studies suggest that PDEVs may serve as a natural integrative platform for bioactive components, exerting therapeutic effects through multi-target and synergistic actions. The following section provides a systematic summary of these potential mechanisms. This includes key findings from RA models discussed in Section *2.4*, as well as insights drawn from studies of other disease models that may also apply to RA.

### 3.1 Immunomodulatory effects

The immunomodulatory potential exhibited by PDEVs is considered one of their core mechanisms that may enable future intervention in autoimmune diseases (43, 68). As shown in Section *2.4*, in RA models, the therapeutic effects of various PDEVs are closely associated with their modulation of macrophage polarization states.

At the direct regulatory level, studies indicate that PDEVs can interact with specific receptors on immune cell surfaces via their membrane components or regulate cell phenotype and function following internalization (68, 81). In RA models, synovial macrophages are the key target, and their regulatory mechanisms have been detailed (see Section *2.4*). These mechanisms have also been extensively explored in other models. For instance, ginseng-derived PDEVs have been shown to influence macrophage polarization, thereby regulating T-cell function (68). Lectin proteins identified in garlic-derived PDEVs can mediate biological effects by binding to the CD98 glycoprotein on macrophage surfaces, providing a clear example of PDEVs directly triggering intracellular signaling via surface ligand-receptor interactions (91). Beyond macrophages, the regulatory effects of PDEVs on dendritic cells (DCs) are also crucial (85, 87). For instance, PDEVs derived from *Petasites japonicus* can promote the expression of co-stimulatory molecules and MHC-II on DCs, thereby enhancing their antigen-presenting capacity (92). In inflammatory models, PDEVs from broccoli can suppress the overactivation of DCs, suppress pro−inflammatory cytokine production and enhance regulatory T−cell induction, facilitating immune tolerance (43, 93). Furthermore, PDEVs can also directly affect T-cell function; for example, celery-derived PDEVs have been confirmed to inhibit the phosphorylation of signaling molecules downstream of the T-cell receptor, directly suppressing T-cell activation (94). At the indirect regulatory level, orally administered PDEVs can exert their effects through the intestinal mucosal immune system (81). For example, Zhang et al. (81) observed that orally administered orange-derived PDEVs can accumulate in the ileocecal region and are specifically taken up by local CD4+ T cells, CD11b+ dendritic cells, and CD11c+ macrophages. In this model, dexamethasone-loaded vesicles suppressed lymphocyte activation in intestinal Peyer’s patches and reduced T-cell activation markers. Such intestinal immunomodulation exerted systemic effects, mitigating distal renal pathology in a mouse model of IgA nephropathy. These findings support the hypothesis that oral PDEVs may modulate intestinal immunity to induce systemic immunoregulatory effects.

In summary, existing research suggests that PDEVs may modulate the immune system via potential pathways involving “direct cellular interactions” and the “indirect regulatory”. However, much of the relevant evidence originates from non-RA-specific models or *in vitro* experiments. The precise cellular targets within the complex microenvironment of RA joints and the specific mechanisms of action of their key active components still require in-depth validation using more rigorous RA disease models.

### 3.2 Anti-inflammatory effects

Research in Section *2.4* has confirmed that PDEVs can effectively suppress key pro-inflammatory factors (such as TNF-α, IL-1β, and IL-6) and inflammatory mediators (including COX-2 and iNOS) in RA models, establishing their fundamental anti-inflammatory properties. Studies in broader inflammatory disease models have also characterized the anti-inflammatory effects of plant-derived exosomes.

Ginger-derived PDEVs have been shown to significantly downregulate the expression of key pro-inflammatory mediators, including NF-κB, TNF-α, IL-8, and IL-1β, in a lipopolysaccharide-induced inflammatory model using intestinal Caco-2 cells. This function is associated with their endogenous miRNA components (45). In breast cancer model, tea-derived PDEVs demonstrate systemic anti-inflammatory effects, primarily by targeting pathways such as NF-κB and MAPK, thereby reducing levels of cytokines including TNF-α, IL-1β, IL-6, and IL-17 (42). Additionally, many PDEVs exhibit a network-based regulatory dimension in their anti-inflammatory action. For example, in a colitis model, turmeric-derived PDEVs effectively inhibit the phosphorylation and nuclear translocation of NF-κB p65. They also significantly upregulate the expression of the antioxidant gene heme oxygenase-1, suggesting that PDEVs may restore the homeostasis between inflammation and oxidative stress (83). These findings suggest that PDEVs may represent a class of natural nanodrug systems with broad-spectrum anti-inflammatory potential, although direct evidence in RA patients remains lacking.

### 3.3 Antioxidant activity

Oxidative stress is a central component in the pathological process of RA. The excessive accumulation of reactive oxygen species resulting from oxidative stress can directly damage joint tissues and amplify inflammatory responses. As natural “nanocarriers” of the plant antioxidant defense system, PDEVs primarily exert protective effects by directly scavenging ROS and activating the host’s endogenous antioxidant pathways.

PDEVs exhibit antioxidant properties through both direct ROS scavenging and activation of endogenous antioxidant pathways. On one hand, PDEVs from plants such as strawberry and lemon are rich in antioxidants including vitamin C, ascorbic acid, citric acid, and glutathione, enabling direct, dose−dependent neutralization of hydrogen peroxide−induced ROS and cytoprotection (32, 95, 96). On the other hand, PDEVs can activate intracellular antioxidant defenses. For instance, tea−derived PDEVs reduce ROS accumulation and upregulate HO−1 in macrophages (42). Among the reported mechanisms of action, the Nrf2/ARE signaling pathway represents a widely studied direction. Carrot-derived PDEVs can upregulate the expression of the transcription factor Nrf2 and its downstream target genes (HO-1, NQO-1) in H9C2 cardiomyocytes (97). Ginger-derived PDEVs have also been confirmed to promote the nuclear translocation of Nrf2 in hepatocytes (98). These studies suggest that activation of the Nrf2 pathway is an important mechanism through which certain PDEVs exert their antioxidant effects. However, the complete signaling network by which PDEVs regulate the antioxidant system remains unclear, and they may function through the coordinated action of multiple pathways, warranting further investigation in future research.

### 3.4 Bone and cartilage protection

PDEVs demonstrate potential for multi-targeted therapeutic intervention in maintaining bone and cartilage homeostasis, with effects encompassing the promotion of anabolic processes and the inhibition of pathological catabolism.

In terms of bone metabolism, lemon-derived PDEVs have been confirmed to significantly promote the osteogenic differentiation of mesenchymal stromal cells and enhance type I collagen synthesis. This effect is attributed to the co-delivery of vitamin C and citrate (71), suggesting that PDEVs possess dual potential in regulating bone formation and potentially influencing osteoclast activity. Regarding cartilage protection, grapefruit-derived PDEVs rebalance chondrocyte metabolism by upregulating anabolic markers and suppressing catabolic factors, while also enhancing chondrocyte migration to support tissue repair (47). In summary, the distinct bone and cartilage protective mechanisms of PDEVs from different plant sources suggest the potential for developing integrated, multifunctional therapeutic platforms against RA destruction through combinatorial or engineering strategies.

### 3.5 The “Cross-Kingdom Regulation” hypothesis

The “Cross-Kingdom Regulation” hypothesis posits that PDEVs can deliver their endogenous bioactive molecules into mammalian cells, thereby regulating host cell gene expression and function. This process is thought to depend particularly on the plant-derived small RNAs carried by PDEVs. Currently, experimental evidence at multiple levels supports this hypothesis.

Research on perilla-derived PDEVs provides direct validation for cross-kingdom regulation. The study not only identified a key plant miRNA within PDEVs but also confirmed that this miRNA can be taken up by mammalian cells. By targeting the conserved HSP90 protein in host cells, it subsequently inhibits key inflammatory pathways such as NF-κB and IL-17 (53). Further experiments demonstrated that encapsulating a mimic of this miRNA into artificial lipid nanoparticles could recapitulate the core therapeutic effects of PDEVs in a disease model, highlighting the potential of specific plant miRNAs as key active components of PDEVs (53). Systematic omics analysis of the molecules carried by PDEVs offers broader support for this hypothesis. For instance, high-throughput sequencing analysis of coconut water-derived PDEVs identified a large number of plant miRNAs. Bioinformatics prediction revealed that these miRNAs may target key pathways in the human genome related to inflammation and metabolism (99). Similarly, analysis of the miRNA composition in broccoli-derived PDEVs and prediction of their human target genes further support their potential for cross-kingdom regulation (74).

The pathological process of RA involves the abnormal activation of multiple key-signaling pathways, including RANKL and NF-κB. In theory, by screening and utilizing PDEVs to deliver plant RNA molecules that can specifically target these core RA pathways, a novel strategy may be developed for achieving localized and precise gene regulation therapy in the joints.

In summary, the cross-kingdom regulation hypothesis is supported by compelling *in vitro* and omics data, but direct *in vivo* evidence in RA models is still limited. The functional significance of plant miRNAs in mammalian cells remains an area of active investigation, and caution is warranted when extrapolating these findings to RA therapy.

### 3.6 Impact on RA via modulation of gut microbiota and the “gut-joint axis”

The “gut-joint axis” theory provides a crucial perspective for the treatment of autoimmune diseases such as RA, as described in Section 2.4. PDEVs, owing to their oral bioavailability and direct contact with the intestinal environment, demonstrate the potential to indirectly intervene in the progression of RA through this axis. A core mechanism involves the systemic modulation of both the composition and function of the gut microbiota.

Research indicates that oral administration of PDEVs from different plant sources can specifically remodel the structure of the gut microbiota. For example, tea-derived PDEVs can significantly increase the alpha diversity of the gut microbiota, specifically manifested by reducing the Firmicutes/Bacteroidetes ratio, decreasing the abundance of potentially harmful bacteria such as Oscillibacter, and increasing the proportion of beneficial bacterial families like Lachnospiraceae (81). Garlic-derived PDEVs selectively enrich *Akkermansia muciniphila*, a bacterium key to immunomodulation, and may further functionally “train” it. This is supported by the finding that after PDEV treatment, outer membrane vesicles from *Akkermansia muciniphila* gain an enhanced ability to cross physiological barriers and exert anti-inflammatory effects *in vitro* (100). Furthermore, broccoli- and kiwifruit-derived PDEVs exert beneficial effects on the gut microecology by enhancing the activity of short-chain fatty acid-producing bacteria (101) or reducing the proportion of potential pathogenic bacteria such as Proteobacteria (102), respectively. These regulatory effects are thought to be related to selective interactions between specific lipid and miRNA components carried by PDEVs and particular gut bacterial populations (72, 92, 100).

In summary, PDEVs may systematically influence host immune homeostasis and thus potentially alleviate systemic inflammation in RA through multiple pathways. These include remodeling microbiota structure, enhancing gut barrier function, promoting beneficial metabolites, and regulating bacterial vesicle activity. However, clinical translation faces challenges such as source-dependent effect heterogeneity, *in vivo* stability issues, and the need to establish a causal link between microbial shifts and joint improvement, all requiring further validation in rigorous RA models.

## 4 Challenges and engineering strategies for PDEVs

### 4.1 Major challenges

#### 4.1.1 Lack of standardization in extraction methods and product heterogeneity

The bioactivity and stability of PDEVs are highly dependent on their extraction and isolation methods. Currently, one of the most critical challenges in the field is the absence of a unified, standardized separation protocol (103). Different extraction techniques vary in principle, operational conditions, and equipment requirements, leading to significant differences in the particle size distribution, purity, and biological activity of the final product (104). For example, PDEVs preparations obtained solely by differential ultracentrifugation may contain substantial impurities such as non-vesicular proteins, necessitating further purification steps such as density gradient ultracentrifugation (103, 105). This methodological inconsistency-induced product heterogeneity severely hinders the comparison and reproducibility of results across different studies, constituting a primary obstacle to their clinical translation and scalable production.

One practical approach to reduce product heterogeneity is to use combinatorial isolation protocols. Coupling ultracentrifugation with density gradient centrifugation, or integrating polymer−based precipitation with ultracentrifugation, improves both yield and purity. Isolation parameters, such as centrifugal force, duration, temperature, gradient composition, should be explicitly reported to facilitate cross−study comparison (106). Method selection should follow a function−oriented framework, depending on the intended downstream application rather than physical uniformity alone. Finally, domain−specific supplementary guidelines for PDEVs would help standardize protocols (107, 108).

#### 4.1.2 Low yield and scalability challenges in production

The low yield of PDEVs is a major problem that limits their progress from laboratory research to large-scale clinical use. This difficulty comes from two main sources: first, the inherently limited efficiency of plant cells in secreting EVs; second, current common separation methods, such as ultracentrifugation, are not efficient or scalable enough to obtain a high yield of intact PDEVs from complex plant materials (109). Although studies have optimized extraction protocols to enhance product purity and consistency, achieving industrial-scale batch production still necessitates the development of more efficient, cost-effective, and standardized manufacturing processes. Recent research has started to explore pre-treatment methods like physical stimulation with ultraviolet light, which aims to trigger stress responses in plant cells to increase both the amount and biological activity of PDEVs. This provides a novel approach to addressing the yield challenge (59).

#### 4.1.3 Lack of specific markers for identification and characterization

In mammalian EVs research, tetraspanins such as CD63 and CD81 are widely accepted as specific markers. However, a similar marker system for PDEVs has not yet been established. Although some candidate proteins have shown potential in specific studies, their conservation and specificity across different plant species require further validation through large-scale research (110). The absence of specific markers makes it difficult for researchers to accurately identify and quantify the true proportion of PDEVs in their obtained samples and to effectively distinguish between different subtypes. This introduces uncertainty into mechanistic investigations and functional attribution.

To address the lack of specific markers and insufficient characterization, orthogonal characterization is essential. Following MISEV 2023, at least two complementary techniques, such as NTA and TEM, should be used to cross−validate size, concentration, and purity (111). Non−vesicular co−isolated impurities also need assessment. Controls such as metabolite−depleted vesicles help distinguish vesicle−mediated effects from phytochemical bioactivity (106, 112). A more fundamental challenge is the lack of validated plant−specific surface markers. Unlike mammalian EVs, PDEVs currently lack such identifiers. Proteomic and lipidomic profiling across multiple sources should be prioritized to identify conserved signature molecules. Ultimately, building on the MISEV framework, domain−specific supplementary guidelines for the characterization of PDEVs would benefit the field (107, 108).

### 4.2 Engineering strategies for PDEVs

**Figure 4:**
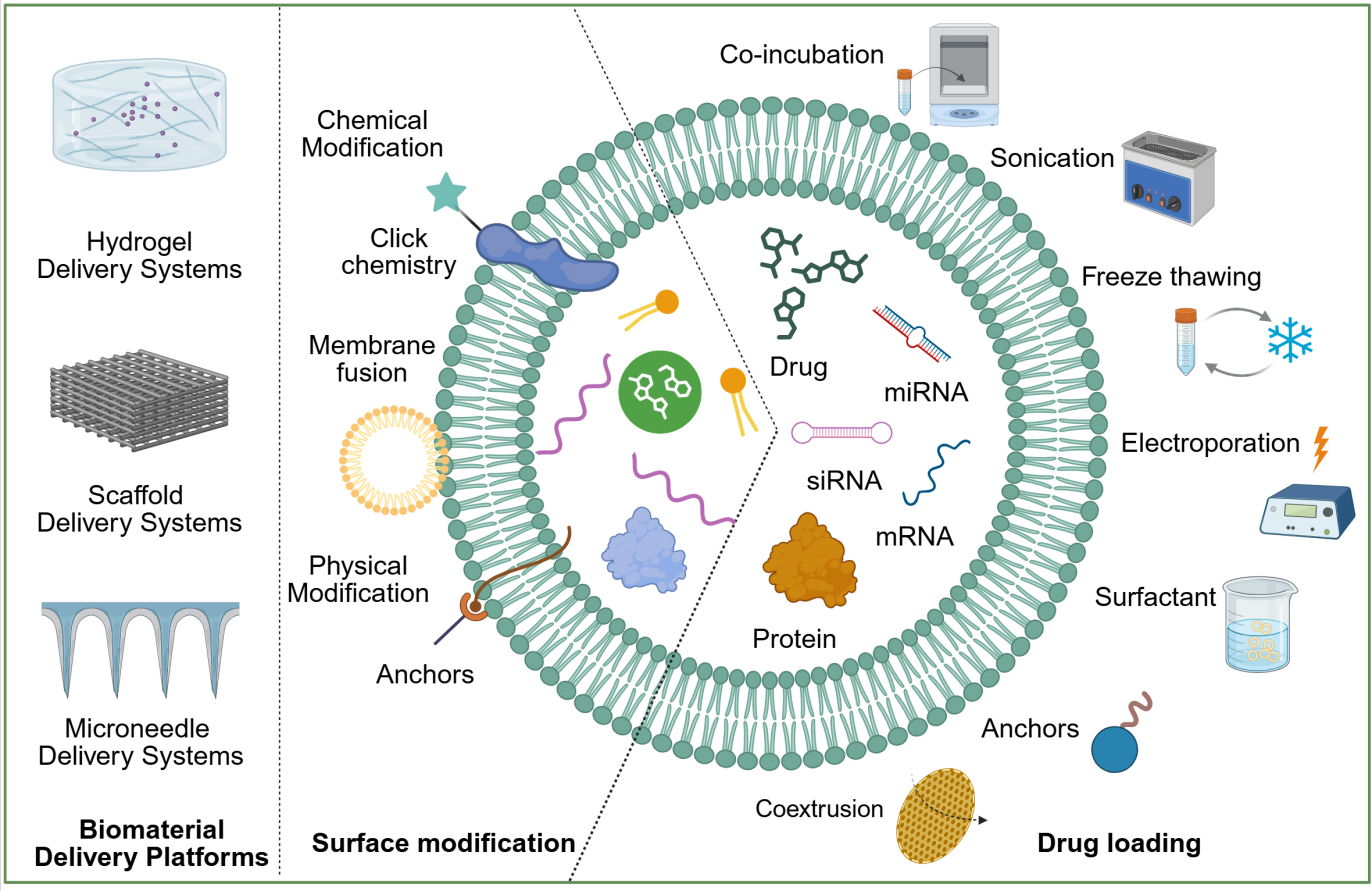
Engineering strategies for therapeutic PDEVs. Created with BioRender.com, accessed on January 9, 2026.

To overcome the inherent limitations of natural PDEVs in targeting, drug-loading capacity, and *in vivo* stability, a series of engineering strategies have been developed (**Figure 4**). These strategies aim to transform PDEVs from natural bioactive carriers into performance-controllable therapeutic platforms, primarily focusing on optimizing drug-loading methods, enhancing targeting capabilities, and integrating with advanced biomaterial systems.

#### 4.2.1 Drug loading

Efficient loading of drugs into PDEVs is fundamental for harnessing their delivery function. Two main technical pathways are currently employed: one involves directly loading drugs into purified natural PDEVs; the other entails extracting key lipid components to reconstruct more homogeneous and controllable plant-derived nanovectors (PDNVs) prior to drug loading (74). Owing to their phospholipid bilayer structure, PDEVs can encapsulate a wide range of drug molecules, including small-molecule drugs, nucleic acids (siRNA, miRNA, mRNA), and proteins. For instance, grapefruit-derived PDEVs can be loaded with HSP70 to exert cytoprotective effects (113); broccoli-derived PDEVs can serve as a delivery platform for exogenous miRNAs (74); and ginger-derived PDEVs can effectively deliver CD98-targeting siRNA for treating intestinal inflammation (114).

Various loading methods have been established for molecules with different properties. Co-incubation is straightforward, relying on concentration gradients for loading, and has been successfully used to load the chemotherapeutic drug doxorubicin into cabbage and celery-derived PDEVs (54, 115). Sonication enhances membrane permeability through transient disturbance, making it suitable for loading macromolecules or hydrophilic drugs, such as co-loading HSP70 protein into tomato- and grapefruit-derived PDEVs (54, 113). Co-extrusion is a common technique for preparing PDNVs, where repeated passage of a plant lipid and drug mixture through polycarbonate membranes yields drug-loaded vesicles with uniform size and high encapsulation efficiency, such as ginger PDNVs loaded with 6-shogaol (116). Additionally, methods like electroporation and freeze-thaw cycles are being explored to optimize the drug-loading efficiency of PDEVs.

This schematic outlines three complementary engineering approaches: (1) Surface Modification through chemical modification including click chemistry and membrane fusion, or physical association using membrane anchors, to enhance targeting and stability; (2) Drug Loading of therapeutic agents such as small molecules, nucleic acids, and proteins via techniques like co-incubation, sonication, electroporation, or coextrusion; and (3) Integration into Biomaterial Delivery Platforms including hydrogels for sustained release, scaffolds for tissue repair, and microneedles for transdermal delivery, to control administration and release kinetics.

#### 4.2.2 Strategies for enhanced targeting

Enhancing the specific accumulation of PDEVs in diseased tissues such as inflamed joints is central to reducing off-target toxicity and improving therapeutic efficacy. Active targeting modification involves chemically conjugating specific ligands (such as RGD peptides, folic acid, and antibody fragments) to the surface of PDEVs. This enables PDEVs to recognize and bind to receptors overexpressed on RA synovium or activated immune cells, thereby achieving “homing” delivery to the lesion site (117, 118). PEGylation serves as a fundamental and effective “stealth” modification strategy. By attaching hydrophilic PEG chains to the PDEV surface, a steric barrier is formed that effectively reduces recognition and clearance by the mononuclear phagocyte system, significantly prolonging their circulation half-life (119). Biomimetic membrane coating technology provides another sophisticated targeting approach. For instance, biomimetic vesicles constructed by coating PDEVs with activated leukocyte membranes can inherit the innate adhesion and migration properties of leukocytes toward inflamed endothelium, demonstrating enhanced accumulation efficiency in inflammatory tissues (90). A more advanced strategy involves vesicle fusion, such as fusing PDEVs with mesenchymal stem cell membrane vesicles that express specific chemokine receptors. These engineered vesicles can actively sense and respond to chemokine gradients within the lesion microenvironment, achieving “chemotactic targeting” and thereby enabling the precise delivery of therapeutic agents to inflammatory sites (120).

#### 4.2.3 Bio-material-based engineered delivery systems

To overcome major *in vivo* delivery limitations of free PDEVs, such as rapid clearance and insufficient targeting, researchers are actively working to integrate PDEVs with various advanced biomaterials. The goal is to construct intelligent delivery systems with spatial positioning control, temporally controlled release, and microenvironment-responsive characteristics.

Hydrogel Delivery Systems, characterized by their highly hydrated three-dimensional polymeric network, excellent biocompatibility, and gentle encapsulation and protection capabilities for bioactive substances, serve as ideal carriers for loading and delivering exosomes (121). Through chemical or physical cross-linking methods, the pore size, mechanical strength, and swelling behavior of hydrogels can be precisely regulated, enabling controlled diffusion release of loaded exosomes or responsive release to specific microenvironmental stimuli (122). In the local treatment of RA in the joint cavity, thermosensitive hydrogels offer distinct advantages. Poloxamer-based formulations, for instance, exist as an injectable sol at room temperature and undergo rapid *in situ* gelation at body temperature upon joint injection. This phase transition effectively prevents vesicle clearance by synovial fluid while providing temporary structural support and lubrication. Studies confirm that exosomes encapsulated in such hydrogels achieve significantly prolonged retention and higher local concentrations compared to intravenously administered free exosomes, substantially extending the therapeutic window (123, 124).

Scaffold Delivery Systems are typically constructed from biodegradable synthetic polymers or naturally derived materials, providing physical support for bone and cartilage repair and guiding tissue regeneration (125). The high porosity and interconnected pore channels of scaffolds not only facilitate the migration and proliferation of host cells but also offer space for the loading and sustained release of exosomes. Animal studies have shown that biodegradable scaffolds loaded with exosomes, when implanted into bone defect areas, can effectively promote new bone formation, improve trabecular bone structure, and enhance bone mineralization, demonstrating the synergistic effects of this system in bone repair (126).

Microneedle Delivery Systems offer a minimally invasive and efficient approach for the transdermal or transmucosal delivery of exosomes (127). Microneedles are typically made of biodegradable materials and are of sufficient length to penetrate the stratum corneum barrier of the skin without reaching pain-sensitive nerves, enabling painless delivery (128). One study co-encapsulated exosome with polydopamine nanoparticles possessing antioxidant and photothermal conversion functions at the tips of dissolvable microneedle patches. This composite microneedle system achieved multiple therapeutic effects, including anti-inflammatory, cartilage protection, and promotion of subchondral bone repair, in an osteoarthritis model (129). In the application of plant exosomes, research has co-loaded ginger exosomes with an analgesic active component into hyaluronic acid microneedles, achieving targeted transdermal drug delivery and significantly increasing the local drug concentration in joints (46).

**Figure 5:**
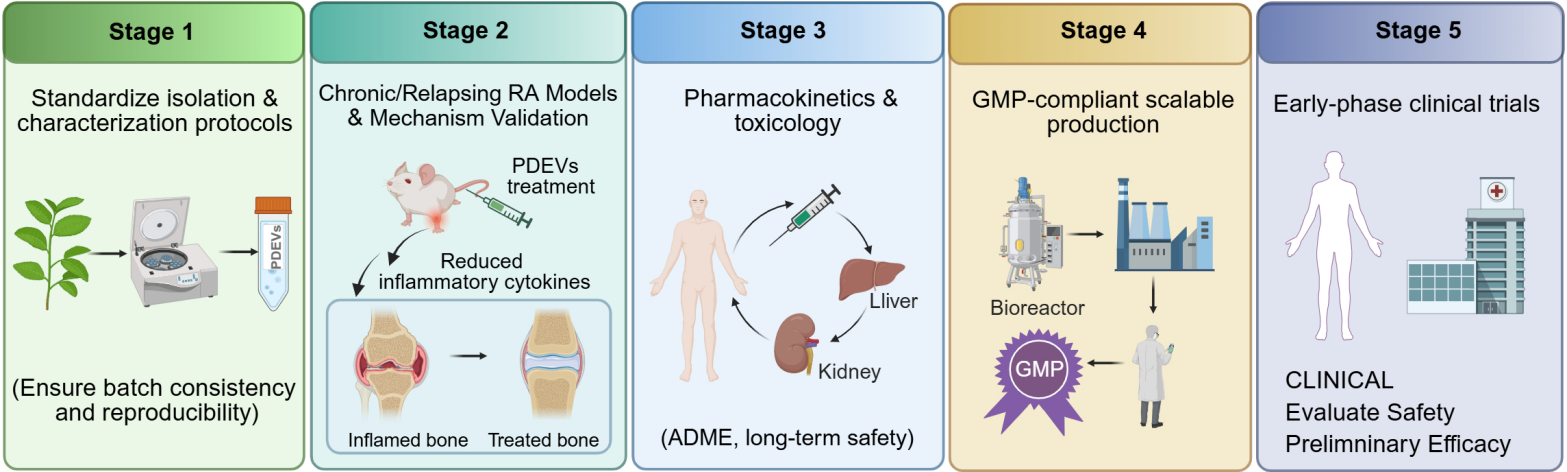
Proposed translational roadmap for PDEVs in RA. The roadmap outlines five sequential stages toward clinical application. Stage 1 focuses on standardization of isolation and characterization protocols to ensure batch consistency and reproducibility. Stage 2 involves validation of efficacy and mechanism in chronic/relapsing RA animal models, with representative indicators such as reduced inflammatory cytokines and bone repair. Stage 3 comprises comprehensive pharmacokinetic and toxicological studies, including ADME (absorption, distribution, metabolism, excretion) profiling and long-term safety assessment. Stage 4 addresses GMP-compliant scalable production, illustrated by a bioreactor system. Stage 5 proposes early-phase clinical trials to evaluate safety and preliminary efficacy. This roadmap highlights key steps toward potential clinical translation. Created with BioRender.com, accessed on April 16, 2026.

In summary, integrating PDEVs into biomaterial delivery systems such as hydrogels, scaffolds, and microneedles represents an effective engineering strategy to overcome delivery challenges such as short retention and uncontrolled release (130). Through intelligent material design, these systems enable precise localization, sustained release, and enhanced protection of PDEVs. This synergistic strategy amplifies their therapeutic potential in modulating the immune microenvironment, suppressing inflammation, and promoting tissue repair. It thereby potentially contributes to a technical foundation for developing more translatable and efficient RA therapies. However, most of these studies remain at the proof-of-concept stage. Key issues such as long-term biocompatibility, consistency between different batches, and the stability of engineered PDEVs during storage and *in vivo* circulation require further investigation before clinical translation. To address these challenges, a stepwise translational roadmap is proposed in **Figure 5**. This roadmap outlines five sequential stages, from standardization and preclinical validation to GMP production and early-phase clinical trials. This framework provides a potential path for future clinical application.

## 5 Conclusions and future perspectives

PDEVs represent a novel class of bioactive nanocarriers with distinctive value in the interventional strategies for RA. This review systematically examines the potential of PDEVs to intervene in the complex pathological network of RA through multi-target and multi-pathway approaches, including mechanisms such as modulating immune cell function, influencing gut microbiota ecology, inhibiting inflammatory cascades, and mitigating oxidative stress. These biological properties, combined with their favorable biocompatibility, low immunogenicity, and stability during oral delivery, position PDEVs as a bridge connecting traditional phytotherapy and modern precision medicine. However, significant hurdles remain in translating these findings from basic research to clinical application. Currently, isolation and preparation techniques for PDEVs lack standardization, leading to considerable variability in the physicochemical properties and bioactivity of products obtained through different methods. This directly compromises the reliability and reproducibility of research outcomes. More critically, understanding of their mechanisms of action *in vivo*, particularly within the local joint microenvironment, remains insufficient. Key issues such as the molecular basis of selective cellular uptake, the intracellular release and action pathways of their cargo, and comprehensive pharmacokinetic profiles require elucidation. These knowledge gaps constrain the rational design and efficient application of PDEVs. In addition, human RA is complex and heterogeneous, and current animal models cannot fully replicate the disease. This may reduce the predictive value of preclinical results. Standardized dosing schedules and pharmacokinetic profiles are not yet available for PDEVs. Their biodistribution, tissue deposition, and elimination pathways remain largely unknown. As a result, uncertainty remains about their efficacy, safety, and therapeutic consistency, which delays the translation of promising preclinical findings into clinical applications. Moreover, PDEVs have not been tested clinically, and robust evidence for their use in RA is still lacking. Therefore, caution is needed when extrapolating the available literature to clinical use.

Despite these limitations, engineering strategies can offer practical pathways to overcome these bottlenecks. Through optimization of drug-loading techniques, modification with targeting molecules, and integration with advanced biomaterial systems, PDEVs have the potential to evolve from natural bioactive carriers into performance-controllable intelligent delivery platforms. Surface engineering can endow them with tissue-specific targeting capabilities, while delivery systems such as hydrogels and microneedles can significantly prolong their retention at lesion sites and achieve controlled release. These technological advances may help address the limitations of natural PDEVs and could create opportunities for more precise and sustained RA treatment.

Looking ahead, advancing PDEVs research will require coordinated efforts across multiple fronts. Firstly, establishing a standardized system for isolation and characterization at the methodological level is essential to ensure comparability and reliability of the research foundation. Secondly, employing systems biology approaches to unravel the complex interaction networks of PDEVs within the RA pathological environment, particularly their crosstalk with immune and bone metabolic systems, is crucial. Concurrently, developing more efficient and safer engineering solutions and conducting comprehensive evaluations in disease models that more closely mimic clinical reality will be critical steps toward translational application. Ultimately, only through well-designed clinical studies to verify their safety and efficacy can this promising novel therapeutic strategy be translated into tangible benefits for patients.

In summary, PDEVs research lies at the intersection of traditional phytotherapy and modern nanotechnology. Despite persistent challenges in standardization, mechanistic understanding, and clinical translation, continued in-depth research and the advancement of engineering technologies hold promise for PDEVs as an innovative RA therapy. This could not only enrich existing therapeutic strategies but also open new avenues for intervention in other autoimmune diseases.
